# A prediction model for the 5-year, 10-year and 20-year mortality of medullary thyroid carcinoma patients based on lymph node ratio and other predictors

**DOI:** 10.3389/fsurg.2022.1044971

**Published:** 2023-01-13

**Authors:** Yanhua An, Jingkai Lu, Mosheng Hu, Qiumei Cao

**Affiliations:** ^1^ Department of General Practice, Beijing Tongren Hospital Affiliated to Capital Medical University, Beijing, China; ^2^ Department of Emergency Medicine, The 305th Hospital of PLA, Beijing, China; ^3^ Department of Otolaryngology, Civil Aviation Medical Assessment Institute, Civil Aviation Medicine Center, CAAC, Beijing, China

**Keywords:** medullary thyroid carcinoma, lymph node ratio, overall survival, cause-specific survival, SEER

## Abstract

**Aim:**

To explore the predictive value of lymph node ratio (LNR) for the prognosis of medullary thyroid carcinoma (MTC) patients, and constructed prediction models for the 5-year, 10-year and 20-year mortality of MTC patients based on LNR and other predictors.

**Methods:**

This cohort study extracted the data of 2,093 MTC patients aged ≥18 years undergoing total thyroidectomy and neck lymph nodes dissection. Kaplan-Meier curves and log-rank tests were performed to compare survival curves between LNR < 15% group and LNR ≥ 15% group. All data was divided into the training set (*n* = 1,465) and the testing set (*n* = 628). The random survival forest model was constructed in the training set and validated in the testing set. The area under the curve (AUC) was employed for evaluating the predictive ability of the model.

**Results:**

The 5-year, 10-year and 20-year overall survival (OS) and cause-specific survival (CSS) of MTC patients with LNR <15% were higher than those with LNR ≥15%. The OS was 46% and the CSS was 75% after 20 years' follow-up. The AUC of the model for the 5-year, 10-year, and 20-year OS in MTC patients was 0.878 (95%CI: 0.856–0.900), 0.859 (95%CI: 0.838–0.879) and 0.843 (95%CI: 0.823–0.862) in the training set and 0.845 (95%CI: 0.807–0.883), 0.841 (95%CI: 0.807–0.875) and 0.841 (95%CI: 0.811–0.872) in the testing set. In the training set, the AUCs were 0.869 (95%CI: 0.845–0.892), 0.843 (95%CI: 0.821–0.865), 0.819 (95%CI: 0.798–0.840) for the 5-year, 10-year and 20-year CCS in MTC patients, respectively. In the testing set, the AUCs were 0.857 (95%CI: 0.822–0.892), 0.839 (95%CI: 0.805–0.873) and 0.826 (95%CI: 0.794–0.857) for the 5-year CCS, 10-year CCS and 20-year CCS in MTC patients, respectively.

**Conclusion:**

The models displayed good predictive performance, which might help identify MTC patients might have poor outcomes and appropriate interventions should be applied in these patients.

## Introduction

Medullary thyroid carcinoma (MTC) is a kind of thyroid cancer neuroendocrine tumor (NET) arising from the thyroid parafollicular C-cells (1). Among them, 75% of the cases are sporadic (75% of cases) and 25% of them are in a hereditary pattern (2). MTC is a rare form of thyroid cancers with the prevalence of 1 to 10% in all thyroid cancers (3). The mean survival of MTC patients was about 8.6 years and the 10-years survival rates varied from 69 to 89% (4). Previous studies indicated that the prognosis of MTC might be correlated with various factors such as gender, age at diagnosis, lymph node metastases, and treatments (5). To deep explore the related predictors for MTC prognosis is essential for the prevention of poor outcomes in those patients.

Lymph node ratio (LNR) is defined as the number of positive lymph nodes divided by the number of lymph nodes resected, which is widely accepted as a potential prognostic factor in tumors (6–8). Renaud et al. also indicated that LNR had superior performance over number of lymph node involved for the prognostic prediction in colorectal carcinoma (9). LNR is an index reflecting the extent of tumor, which harbors a high value suggesting the tumor stage and prognosis (10). LNR is calculated based on the number of resected lymph nodes, which also reflects the extent of surgery (11). LNR is an index not only representing the burden of tumors but also reflecting the surgical and pathological quality standards (12). Currently, there were studies exploring the predictive ability of the number of positive lymph nodes on the prognosis of MTC (13). A multi-center cohort study also reported the association of LNR and the prognosis of MTC patients (14). The predictive value of LNR for the short, and long term outcomes of MTC patients was still unclear.

Random forests model is a novel machine learning technique attracting increasing attention due to its excellent performance and great flexibility in handling all types of data, including “big data.” (15). Random forests with a survival outcome are known as random survival forests, which can obtain the cumulative hazard functions for each tree based on 36.8% of the data that were not applied to grow it for greater accuracy and averaged across trees to get a final forest cumulative hazard function for each observation (16). Previously, random survival forests model was widely employed to analyze survival problems with great success such as overall survival after esophagectomy (17) or survival of patients with breast cancer (18). Compared to traditional Kaplan-Meier and Cox proportional hazards regression analyses, random survival forests model requires less-restrictive assumptions and is able to accommodate various kinds of predictors and interactions among them (19). Random survival forests can analyze high-dimensional data sets when the predictors are more than the sample size of the data (20). Random survival forests modeling can provide its own internal generalization error estimate as well as measures of variable importance for each variable included in the model. To our knowledge, there was no study using random survival forests for predicting the survival of MTC patients.

The purpose of this study was to explore the predictive value of LNR for the prognosis of MTC patients, and constructed a prediction model for the 5-year, 10-year and 20-year mortality of MTC patients based on lymph node ratio and other predictors. The findings of our study might provide a tool for identifying MTC patients at high risk of mortality, and offer timely interventions for improving the outcomes of these patients.

## Methods

### Study design and population

In total, this cohort study extracted the data of 2,701 MTC patients aged ≥18 years who underwent total thyroidectomy and neck lymph nodes dissection from SEER database (www.seer.cancer.gov) (SEER 9 Registries Dataset, SEER 13 Registries Dataset and SEER 18 Registries Dataset). SEER database is a freely-accessed cancer database involving approximately 28% of the US population (21). Patients data including demographic data, primary tumor data, regional nodal data, vital status, and survival were available in SEER database. SEER-Stat (version 8.3.5, National Cancer Institute, USA) was applied for filtering and collecting the data of eligible patients. MTC patients were identified from SEER database based on the International Classification of Diseases for Oncology (ICD-O): 8345/3. After excluding duplicate patients, those with overall survival <1 month and LNR could not be calculated, 2,093 patients were finally analyzed.

### Potential predictors

Gender, age (years), race [White, Black, others (American Indian/AK Native, and Asian/Pacific Islander) or unknown], marital status (married, single, others or unknown), year of diagnosis (1975–1979, 1980–1989, 1990–1999, 2000–2009 or 2010–2017), tumor size (<40 mm, ≥40 mm or unknown), stage (localized, regional, distant or unknown), radiation (yes or no), chemotherapy (yes or no), regional nodes positive (0, 1–10 or >10), and regional nodes examined (0, 1–10 or >10).

### Main variable and outcome variables

LNR was the main variable in the current study, which was defined as the number of metastatic lymph nodes divided by the number of lymph nodes resected, also known as the lymph node yield. The cutoff value of LNR was set as 15% based on a previous study (14).

Overall survival (OS) and cause-specific survival (CSS) were outcome variables in this study. OS refers to the period from the time of surgery to the death of any cause or the date of the last follow-up, while CSS was calculated from the time of operation to the date of cancer-related death or the time of last follow-up. Whether the patients were alive or death, as well as whether patients were died of other causes or MTC were identified in 5 years' follow-up, 10 years' follow-up and 20 years' follow-up.

### Establishment of the random survival forest model

Random survival forest model was an ensemble tree method for the analysis of OS of MTC patients. Trees in a survival forest are grown through a two-step randomization process randomly. First, each tree is grown in a randomly drawn bootstrap sample (training set). Second, random variable selection is employed when growing the tree. At each split, a new random subset of candidate variables is selected. The bootstrap sample, including for each tree a random subset of the study population, can be seen as the root of the tree. During the tree-growing process, the root is split into two branches. The branch is split using the variable, from the randomly selected subset of candidate variables, that indicates the largest survival difference between daughter branches. Averaging over trees in combination with the randomization used in growing a tree creates an ensemble of independent trees that form the random survival forest.

### Statistical analysis

Quantitative variables were described in terms of mean ± standard deviation (mean ± SD) or *M* (Q_1_, Q_3_), while qualitative variables were displayed as *n* (%). *t* test or Kruskal-Wallis test were selected for comparisons of quantitative variables and Chi-square test or Fisher's exact probability method were applied for comparing differences of qualitative variables between groups. Kaplan–Meier curves and log-rank tests were performed to compare survival curves between LNR <15% group and LNR ≥15% group. All data was divided into the training set (*n* = 1,465) and the testing set (*n* = 628). The random survival forest model was constructed in the training set through the randomForestSRC package, and 500 binary survival trees were generated each time. The data in the testing set were used for validate the results in the training set. The area under the curve (AUC) was employed for evaluating the predictive ability of the model. *P *< 0.05 was set as statistical difference. R 4.1.1 (Institute for Statistics and Mathematics, Vienna, Austria) was utilized for data analysis.

## Results

### The baseline characteristics of participants

**Figure 1:**
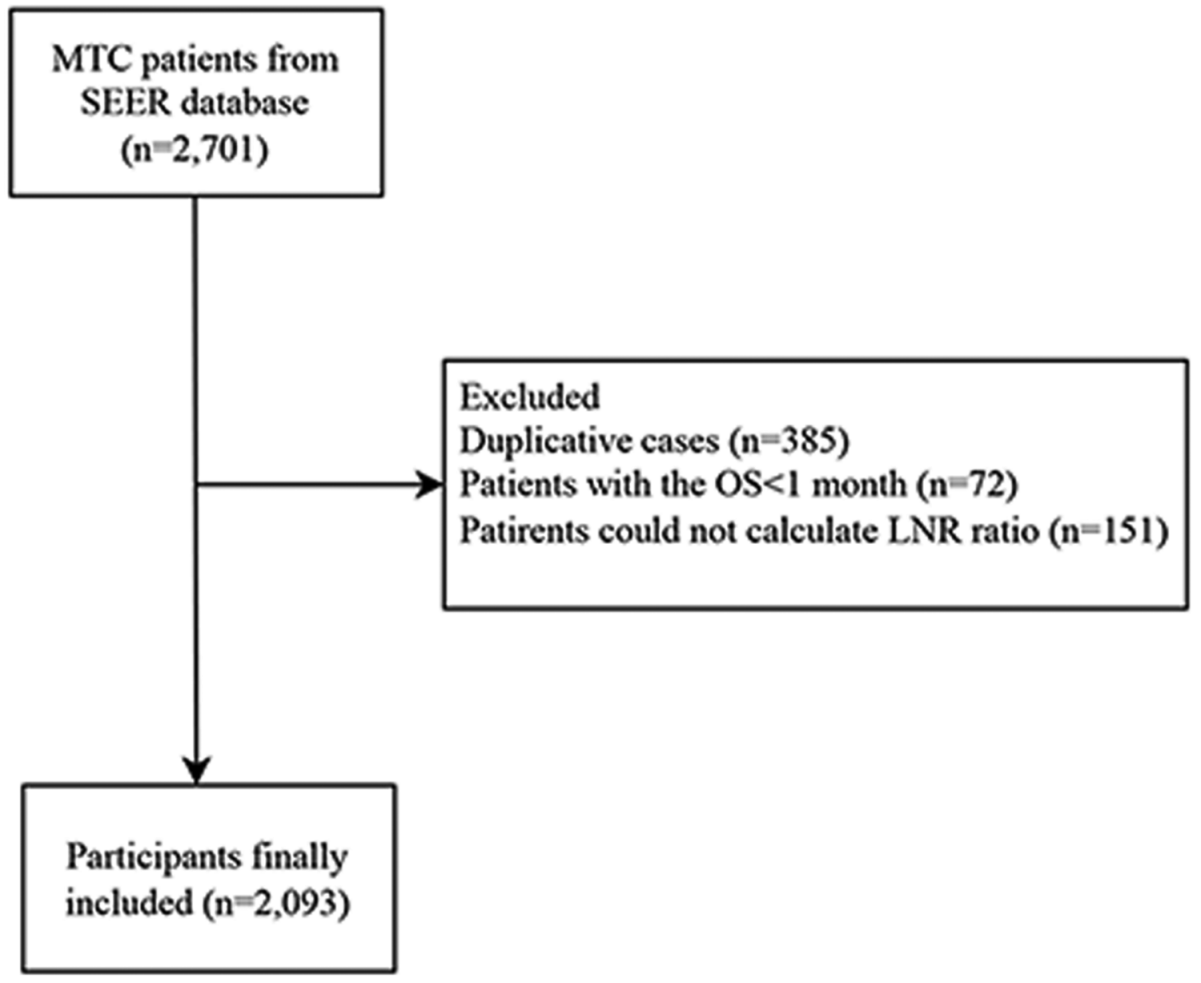
The screen process of the participants.

This study collected the data of 2,701 MTC patients. After removing duplicative cases (*n* = 385) and excluding patients with the OS < 1 month (*n* = 72) and those could not calculate LNR ratio (*n* = 151), 2,093 patients were finally included. The screen process was shown in Figure 1. All data were classified into the training set (*n* = 1,465) and the testing set (*n* = 628).

Among all the participants, 38% of them were males and 61% of them were females. The median age of all patients were 53 years old. As for tumor size, the tumor size of 1,188 patients were <40 mm, accounting for 56.8% of all participants, and the tumor size of 216 people were ≥40 mm, accounting for 10.3% of all patients. 371 subjects received radiation and 90 patients received chemotherapy. 49.3% people without positive regional nodes, 17.5% had 0–10 positive regional nodes and 5.0% patients had >10 positive regional nodes. There were 1,697 people with the LNR <15% and 396 subjects with the LNR ≥15% (Table 1).

**Table 1:** The baseline characteristics of the participants.

Variable	Total (*n* = 2,093)	Training set (*n* = 1,465)	Testing set (*n* = 628)	*P*
Sex				0.371
Male	812 (38.8)	578 (39.5)	234 (37.3)	
Female	1,281 (61.2)	887 (60.5)	394 (62.7)	
Age	53.00 (39.00, 66.00)	53.00 (39.00, 66.00)	53.00 (39.00, 65.00)	0.970
Age Group				0.822
<50	966 (46.2)	679 (46.3)	287 (45.7)	
≥50	1,127 (53.8)	786 (53.7)	341 (54.3)	
Race				0.181
White	1,860 (88.9)	1,293 (88.3)	567 (90.3)	
Black	116 (5.5)	80 (5.5)	36 (5.7)	
Other (American Indian/AK Native, Asian/Pacific Islander)	97 (4.6)	75 (5.1)	22 (3.5)	
Unknown	20 (1.0)	17 (1.2)	3 (0.5)	
Marriage Status				0.876
Married	1,307 (62.4)	923 (63.0)	384 (61.1)	
Single	328 (15.7)	226 (15.4)	102 (16.2)	
Others	383 (18.3)	265 (18.1)	118 (18.8)	
Unknown	75 (3.6)	51 (3.5)	24 (3.8)	
Year of diagnosis				0.136
1975–1979	272 (13.0)	178 (12.2)	94 (15.0)	
1980–1989	560 (26.8)	405 (27.6)	155 (24.7)	
1990–1999	834 (39.8)	581 (39.7)	253 (40.3)	
2000–2009	405 (19.4)	282 (19.2)	123 (19.6)	
2010–2017	22 (1.1)	19 (1.3)	3 (0.5)	
Tumor size group				0.924
<40 mm	1,188 (56.8)	831 (56.7)	357 (56.8)	
≥40 mm	216 (10.3)	149 (10.2)	67 (10.7)	
Unknown	689 (32.9)	485 (33.1)	204 (32.5)	
Stage				0.850
Localized	1,069 (51.1)	741 (50.6)	328 (52.2)	
Regional	674 (32.2)	474 (32.4)	200 (31.8)	
Distant	268 (12.8)	193 (13.2)	75 (11.9)	
Unknown	82 (3.9)	57 (3.9)	25 (4.0)	
Radiation				0.177
No	1,722 (82.3)	1,194 (81.5)	528 (84.1)	
Yes	371 (17.7)	271 (18.5)	100 (15.9)	
Chemotherapy				0.725
No	2,003 (95.7)	1,404 (95.8)	599 (95.4)	
Yes	90 (4.3)	61 (4.2)	29 (4.6)	
Regional nodes positive				0.933
0	1,032 (49.3)	716 (48.9)	316 (50.3)	
1–10	367 (17.5)	259 (17.7)	108 (17.2)	
>10	105 (5.0)	73 (5.0)	32 (5.1)	
Unknown	589 (28.1)	417 (28.5)	172 (27.4)	
Regional nodes examined				0.771
0	1,307 (62.4)	917 (62.6)	390 (62.1)	
1–10	401 (19.2)	284 (19.4)	117 (18.6)	
>10	385 (18.4)	264 (18.0)	121 (19.3)	
LNR				0.778
<15%	1,697 (81.1)	1,185 (80.9)	512 (81.5)	
≥15%	396 (18.9)	280 (19.1)	116 (18.5)	
OS				0.763
Alive	962 (46.0)	677 (46.2)	285 (45.4)	
Dead	1,131 (54.0)	788 (53.8)	343 (54.6)	
CSS				0.849
Alive or dead of other causes	1,569 (75.0)	1,096 (74.8)	473 (75.3)	
Dead of this cancer	524 (25.0)	369 (25.2)	155 (24.7)	

OS, overall survival; CSS, cause-specific survival; LNR, lymph node ratio.

### Construction of the prediction model for the 5-year, 10-year and 20-year Os and CSS of MTC patients

The predictors for the 5-year, 10-year and 20-year OS and CSS of MTC patients were explored based on the variable importance using random forest model. Important variables associated with the 5-year, 10-year and 20-year OS of MTC patients were age, tumor stage, tumor size, radiation, marriage, sex and LNR (Table 2). As for predictors associated with the 5-year, 10-year and 20-year CSS of MTC patients, stage, age, radiation, chemotherapy, tumor size, and LNR were important variables (Table 2).

**Table 2:** The top six important variables associated with the OS and CSS in MTC patients.

Variable	Importance
**OS**	
Age	0.110396
Stage	0.047122
Tumor Size	0.023243
Radiation	0.015358
Marriage	0.014301
Sex	0.013787
LNR	0.011772
**CSS**	
Stage	0.098877
Age	0.041038
Radiation	0.024482
Chemotherapy	0.017040
Tumor size	0.016626
LNR	0.016216
Sex	0.010854

OS, overall survival; CSS, cause-specific survival; MTC, medullary thyroid carcinoma; LNR, lymph node ratio.

**Figure 2:**
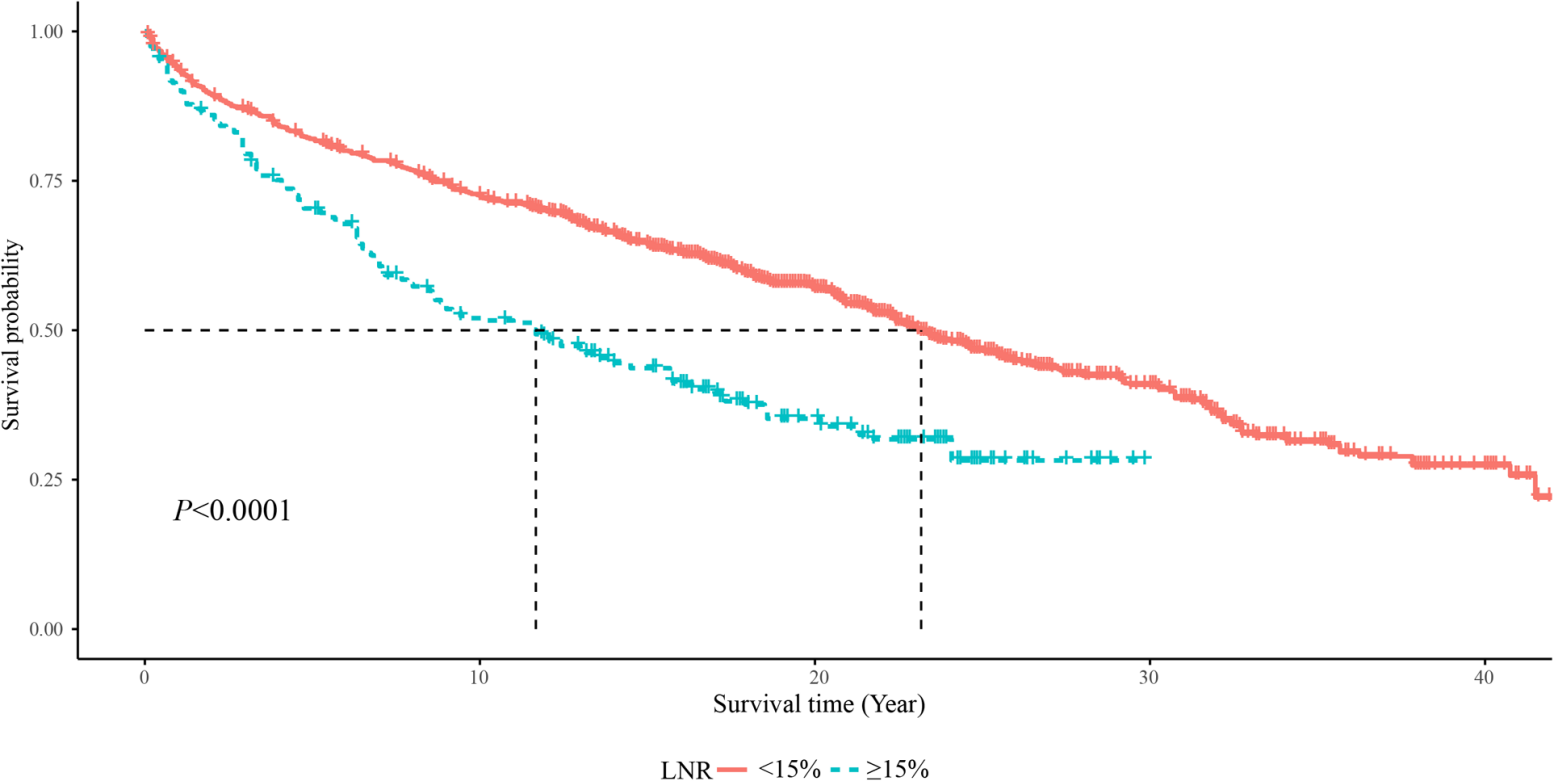
The Kaplan–Meier curve of the OS in LNR <15% and LNR ≥15% groups.

**Figure 3:**
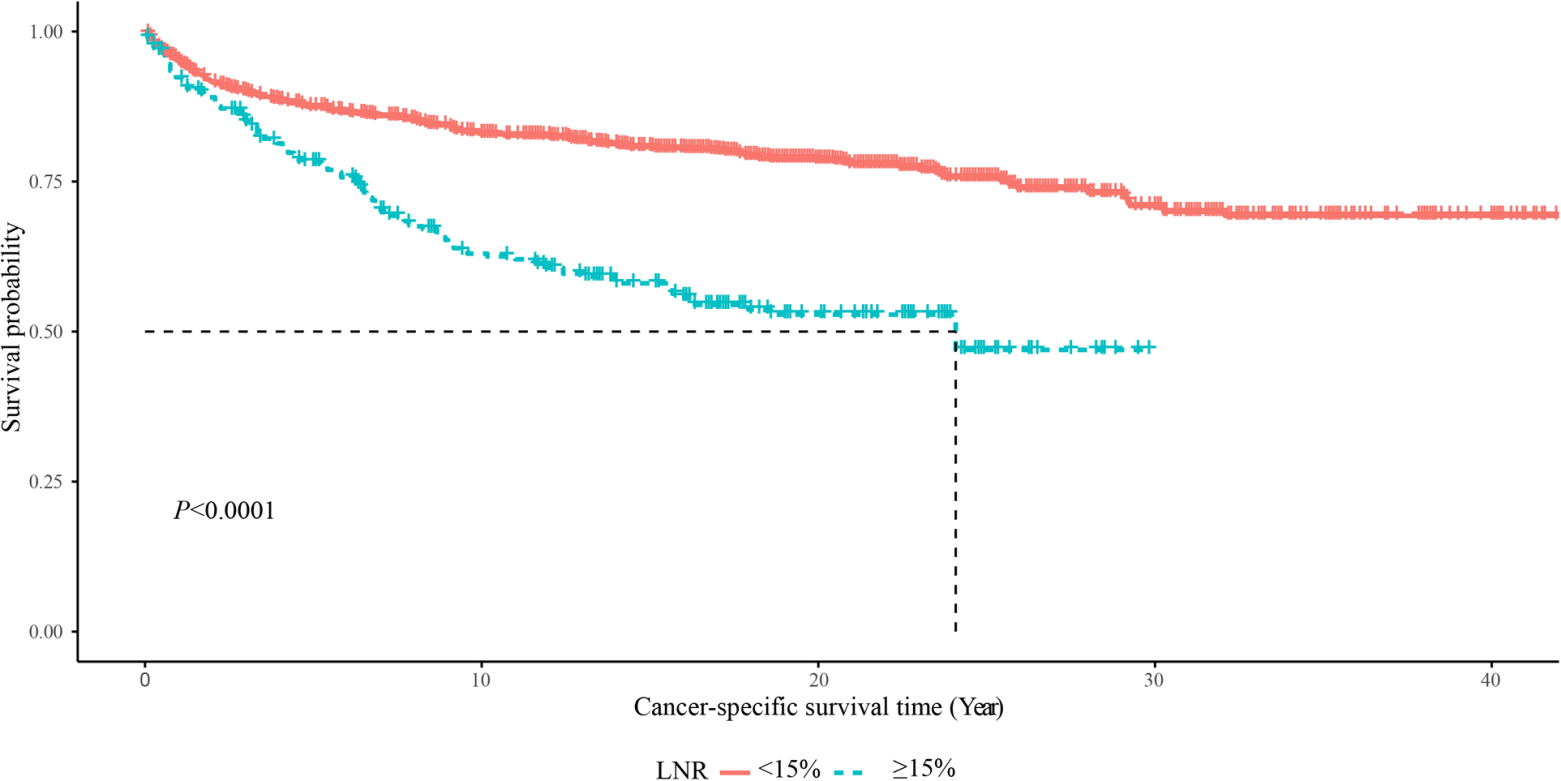
The Kaplan–Meier curve of the CSS in LNR <15% and LNR ≥15% groups.

As displayed in Figure 2, the 5-year OS in the LNR <15% group was about 85%, and in the LNR ≥15% group was about 75%. The 10-year OS was about 75% in the LNR <15% group and 50% in the LNR ≥15% group. The 20-year OS was about 60% in the LNR <15% group and 35% in the LNR ≥15% group. The 5-year, 10-year and 20-year OS of MTC patients with LNR <15% were significant higher than those with LNR ≥15%, respectively. In terms of cause-specific survival, the 5-year (90% vs. 75%), 10-year (85% vs. 65%) and 20-year (80% vs. 50%) CCS in the LNR <15% group were higher than the LNR ≥15% group (Figure 3).

### The predictive value of the random forest model for predicting the 5-year, 10-year and 20-year Os and CSS in MTC patients

The AUC of the model for the 5-year OS in MTC patients was 0.878 (95%CI: 0.856–0.900) in the training set and 0.845 (95%CI: 0.807–0.883) in the testing set (Supplementary Figures S1A, S2A). As for the prediction model for the 10-year OS, the AUC was 0.859 (95%CI: 0.838–0.879) in the training set and 0.841 (95%CI: 0.807–0.875) in the testing set (Supplementary Figures S1B, S2B). The AUC was 0.843 (95%CI: 0.823–0.862) in the training set and 0.841 (95%CI: 0.811–0.872) in the testing set of the prediction model for 20-year OS in MTC patients (Supplementary Figures S1C, S2C). More detailed information of the model was shown in Table 3.

**Table 3:** The predictive ability of the random forest models for the 5-year, 10-year and 20-year OS or CSS in MTC patients.

	Cut-off	Sensitivity (95%CI)	Specificity (95%CI)	PPV (95%CI)	NPV (95%CI)	AUC (95%CI)	Accuracy (95%CI)
Training set							
5-year OS	0.244	0.840 (0.798–0.882)	0.803 (0.780–0.826)	0.517 (0.472–0.562)	0.952 (0.939–0.966)	0.878 (0.856–0.900)	0.810 (0.790–0.830)
10-year OS	0.398	0.717 (0.675–0.758)	0.856 (0.834–0.878)	0.689 (0.648–0.731)	0.871 (0.851–0.892)	0.859 (0.838–0.879)	0.813 (0.793–0.833)
20-year OS	0.559	0.736 (0.702–0.770)	0.806 (0.779–0.833)	0.750 (0.716–0.783)	0.794 (0.766–0.821)	0.843 (0.823–0.862)	0.775 (0.753–0.796)
Testing set							
5-year OS	0.251	0.765 (0.693–0.837)	0.823 (0.789–0.856)	0.534 (0.463–0.606)	0.929 (0.905–0.953)	0.845 (0.807–0.883)	0.811 (0.780–0.841)
10-year OS	0.391	0.686 (0.620–0.753)	0.860 (0.828–0.892)	0.672 (0.605–0.739)	0.868 (0.836–0.900)	0.841 (0.807–0.875)	0.809 (0.778–0.840)
20-year OS	0.508	0.789 (0.741–0.837)	0.762 (0.718–0.806)	0.721 (0.670–0.772)	0.823 (0.781–0.864)	0.841 (0.811–0.872)	0.774 (0.741–0.807)
Training set							
5-year CSS	0.171	0.882 (0.837–0.926)	0.762 (0.739–0.786)	0.374 (0.330–0.417)	0.976 (0.966–0.985)	0.899 (0.879–0.920)	0.779 (0.758–0.800)
10-year CSS	0.282	0.789 (0.741–0.836)	0.798 (0.776–0.821)	0.485 (0.439–0.530)	0.940 (0.926–0.955)	0.872 (0.851–0.894)	0.797 (0.776–0.817)
20-year CSS	0.323	0.783 (0.739–0.827)	0.770 (0.746–0.795)	0.505 (0.462–0.548)	0.923 (0.905–0.940)	0.856 (0.835–0.878)	0.773 (0.752–0.795)
Testing set							
5-year CSS	0.219	0.750 (0.660–0.840)	0.833 (0.802–0.865)	0.423 (0.346–0.501)	0.953 (0.934–0.972)	0.859 (0.818–0.900)	0.822 (0.792–0.852)
10-year CSS	0.306	0.743 (0.663–0.824)	0.837 (0.805–0.869)	0.500 (0.424–0.576)	0.937 (0.915–0.959)	0.848 (0.809–0.888)	0.820 (0.790–0.850)
20-year CSS	0.366	0.683 (0.607–0.760)	0.850 (0.818–0.882)	0.571 (0.496–0.645)	0.902 (0.874–0.929)	0.825 (0.785–0.865)	0.812 (0.782–0.843)

OS, overall survival; CSS, cause-specific survival; MTC, medullary thyroid carcinoma; PPV, positive predictive value; NPV, negative predictive value.

In the training set, the AUCs were 0.869 (95%CI: 0.845–0.892), 0.843 (95%CI: 0.821–0.865), 0.819 (95%CI: 0.798–0.840) for the 5-year CCS, 10-year CCS and 20-year CCS in MTC patients, respectively (Supplementary Figures S3A–C). In the testing set, the AUCs were 0.857 (95%CI: 0.822–0.892), 0.839 (95%CI: 0.805–0.873) and 0.826 (95%CI: 0.794–0.857) for the 5-year CCS, 10-year CCS and 20-year CCS in MTC patients, respectively (Supplementary Figures S4A–C). The sensitivity, specificity, PPV, NPV and accuracy of the models were exhibited in Table 3.

## Discussion

In the present study, the data of 2,093 MTC patients aged ≥18 years who underwent total thyroidectomy and neck lymph nodes dissection were evaluated to identify the predictors for the 5-year, 10-year, 20-year OS and CSS of MTC patients. The data delineated that age, stage, chemotherapy, radiation, tumor size and LNR were associated with 5-year, 10-year, 20-year OS and CSS of MTC patients. The prediction models were established based on these predictors, and the predictive abilities of the models for 5-year, 10-year, 20-year OS and CSS of MTC patients were good. The findings of our study might provide a tool for quickly identifying MTC patients who at high risk of death and timely interventions might be offered in these patients. For patients with old age, high tumor stage, large tumor size, and especially high LNR, more attention should be paid. For the treatments, chemotherapy and radiation might be provided if necessary.

In the present study, we found that LNR was related to the prognosis of patients with MTC. MTC patients with LNR <15% had significantly lower OS and CCS than those with LNR ≥15%. LNR was widely identified as a prognostic factor for MTC patients. A retrospective multicenter study from Rozenblat et al. showed that LNR was an independent factor for disease-free survival of MTC patients (14). Wu et al. identified that LNR >1/3 were more likely to develop progressive disease in patients with MTC (22). Chen et al. conducted a study based on that data of 1,237 MTC patients from the SEER revealed that LNR was a predictor for the prognosis of MTC patients (23). Another study involved in 163 MTC patients reported that LNR were a significant predictor of loco-regional recurrence or persistent disease in MTC (24). In our study, age was also found to be a predictor for the 5-year, 10-year and 20-year OS and CSS of MTC patients. This was allied by a study from Gogna et al., which depicted that increasing age was detrimental to OS of patients with MTC aged ≥45 years (25). Tumor size was another predictor for the 5-year, 10-year and 20-year OS and CSS of MTC patients in this study. Previous studies validated the findings, which showed that larger tumor size might lead to higher risk of tumor recurrence (26) and the tumor size was linked to a reduced survival trend (27). An evaluation of adherence with the 2009 American Thyroid Association Guidelines indicated that the prognosis of MTC patients depends on the stage of the disease at diagnosis (28). Wu et al. also found that MTC patients at stage I, II, and III had more favorable outcomes than those at stage IV (22). These gave support to the results of the current study, which delineated that tumor stage was an important index of the prognosis of MTC patients. In a previous study, the results revealed that stereotactic radiotherapy was an effective treatment for MTC patients (29). Other studies showed controversial results as radiotherapy was found to be effective for local control and whether postoperative radiotherapy affects the survival of MTC patients still lacked convincing evidence (30). The efficacy and safety of chemotherapy in the treatment of MTC was also confirmed in previous studies (31). While another study revealed that surgery and radiation was associated with higher mortality rate in patients in high tumor stage than surgery only (32). Herein, radiation and chemotherapy were predictors for the 5-year, 10-year and 20-year OS and CSS of MTC patients. For MTC patients, appropriate radiation or chemotherapy should be applied to after evaluating the status of patients. For those without proper conditions, radiation or chemotherapy should be performed with caution.

This study established two prediction models for the 5-year, 10-year and 20-year OS and CSS of MTC patients based on the above predictors, respectively. In 2015, Ho et al. constructed a postoperative nomogram for predicting the CSS in MTC patients (33). The nomogram used the predictors including age, gender, pre- and postoperative serum calcitonin, pre- and postoperative carcinoma embryonic antigen, rearranged during transfection mutation status, perivascular invasion, margin status, pathologic T status, pathologic N status, and M status, which showed a concordance index of 0.77. Another prediction nomogram for 5-year OS, and CSS in MTC patients was also established, and the predicted concordance index of OS and CSS was 0.813 and 0.828 in the testing group, respectively (23). In the present study, the AUCs for the 5-year, 10-year and 20-year OS in MTC patients were 0.845, 0.841, and 0.841 and for the 5-year, 10-year and 20-year CCS in MTC patients were 0.857, 0.839 and 0.826in the testing set, respectively. The models presented good discriminative ability for MTC patients with high risk of mortality. In addition, the specificity, NPV and the accuracy of the models were good, indicating the models might provide a tool for quickly identifying MTC patients who might have high risk of death within 5 years, 10 years and 20 years. We have uploaded the model onto the GitHub (https://github.com/caoqiumei/project), and clinicians can freely use our prediction model online. For those who were predicted with high risk of mortality, the clinicians might provide timely treatments and hope to improve the short, and long term outcomes in those patients. Several limitations existed in our study. Firstly, no genetic information could be obtained due to the limitation of data in SEER database, and we could not identify the subtypes of MTC in patients. Secondly, the random survival forest model could only identify the association between variables and outcomes, but the direction of each variable on the outcome was not clear. In this study, we combined the results of our study with Cox's proportional hazard analyses to identify the direction of each variable on the outcome (Supplementary Figures S5, S6) and provide reference for the treatments and interventions in clinic.

## Conclusion

This study constructed prediction models for the 5-year, 10-year, 20-year OS and CSS of MTC patients based on age, stage, chemotherapy, radiation, tumor size and LNR. The models displayed good predictive performance, which might help identify MTC patients might have poor outcomes and appropriate interventions should be applied in these patients.

## Data Availability

Publicly available datasets were analyzed in this study. This data can be found here: SEER database, https://seer.cancer.gov/.
