# Supplementary figures and images for: A prediction model for the 5-year, 10-year and 20-year mortality of medullary thyroid carcinoma patients based on lymph node ratio and other predictors
Source: Front Surg. 2023 Jan 13;9:1044971. doi: 10.3389/fsurg.2022.1044971 (PMC9879301; doi:10.3389/fsurg.2022.1044971)

A

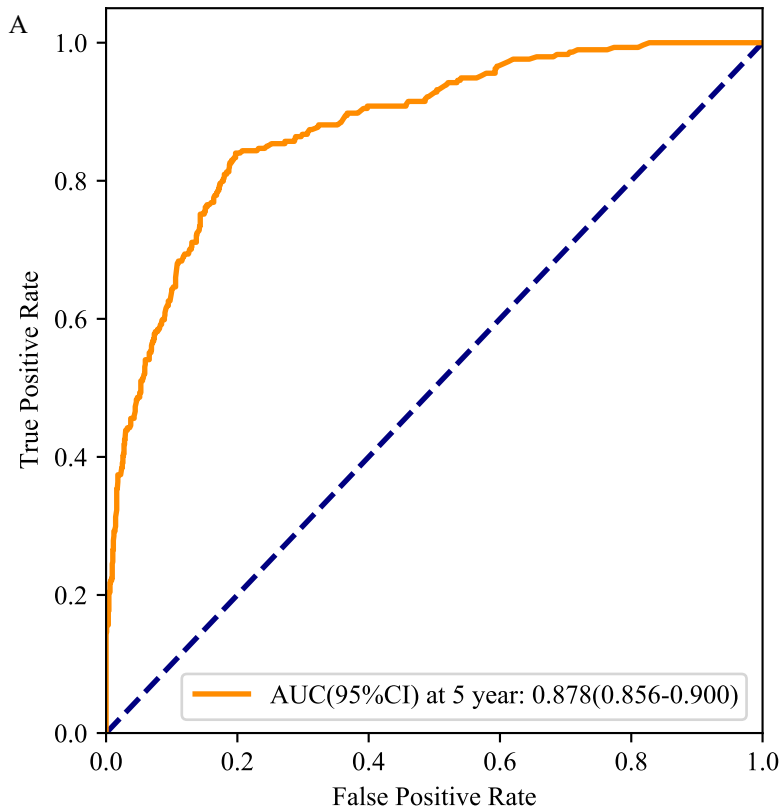

B

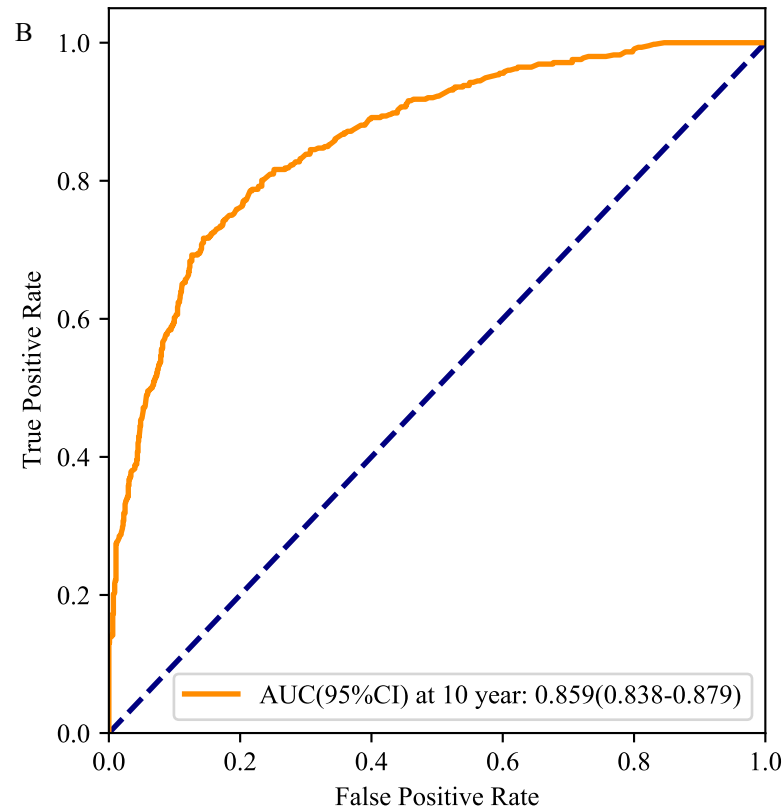

C

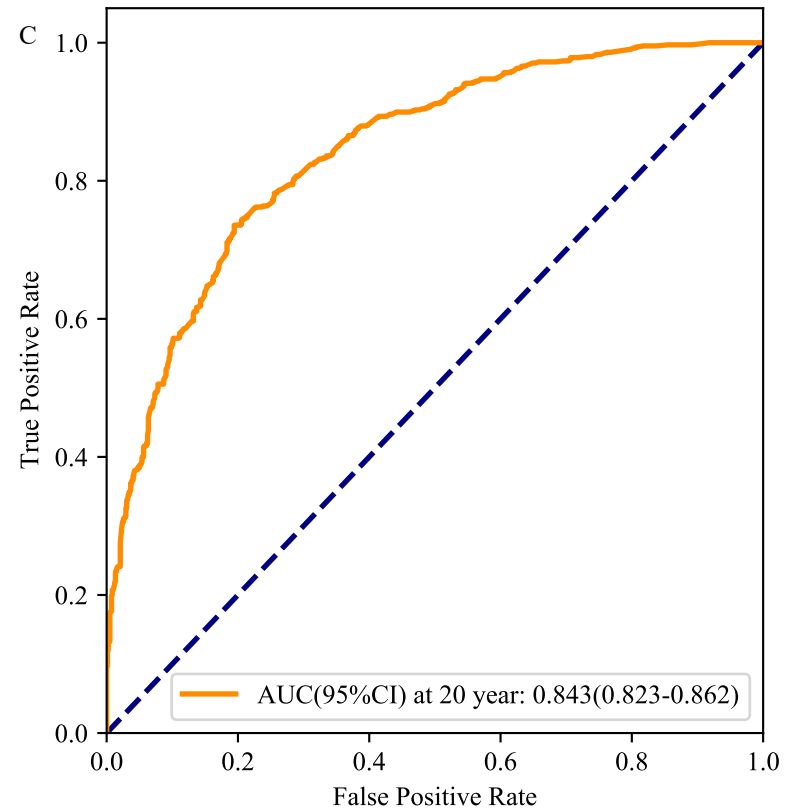

Supplement: Supplementary file 1 [file Datasheet1.pdf]

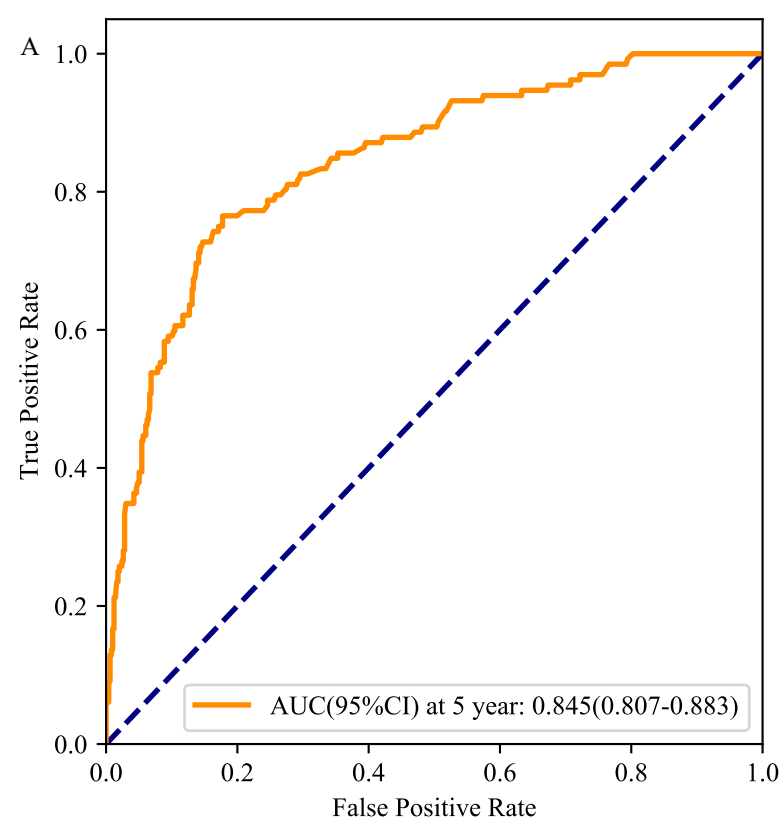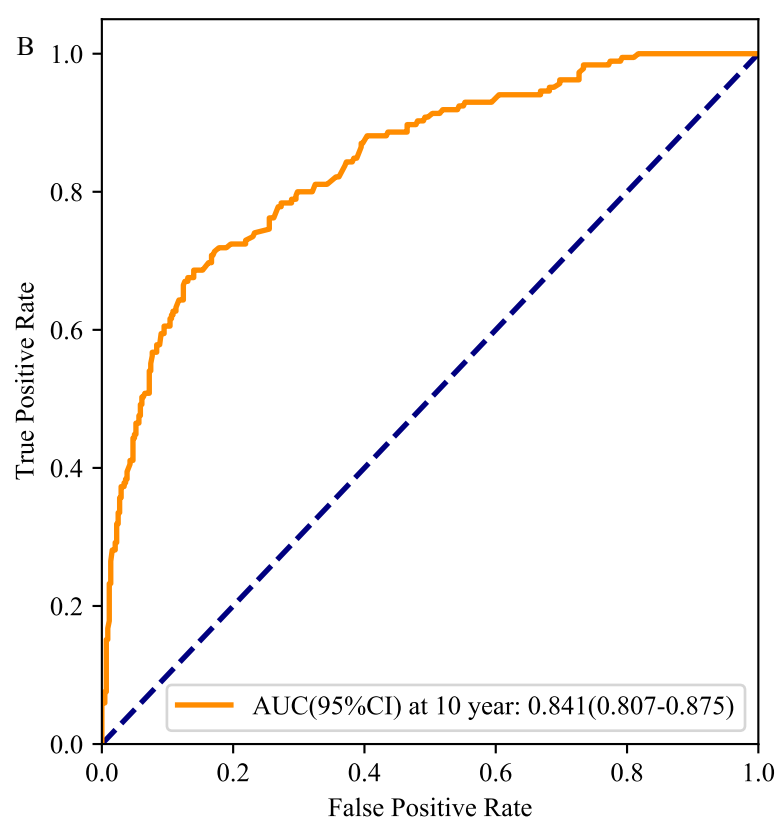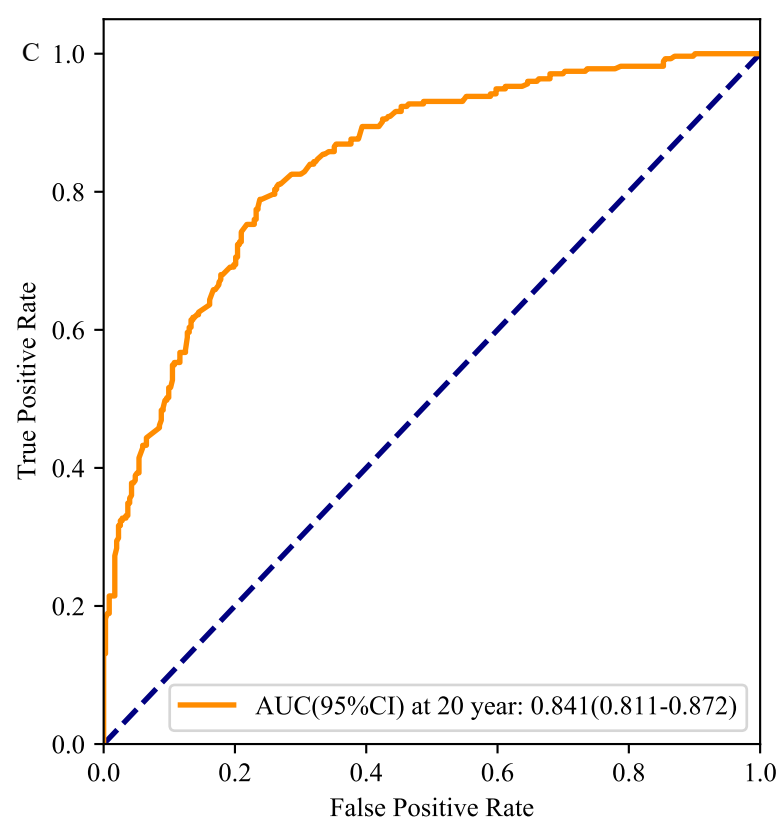

Supplement: Supplementary file 2 [file Datasheet2.pdf]

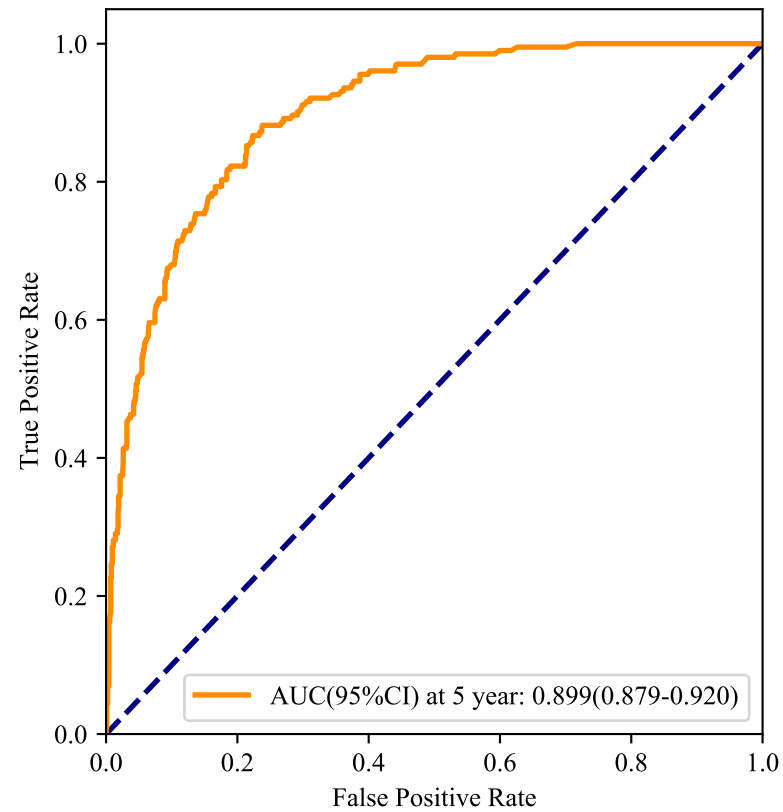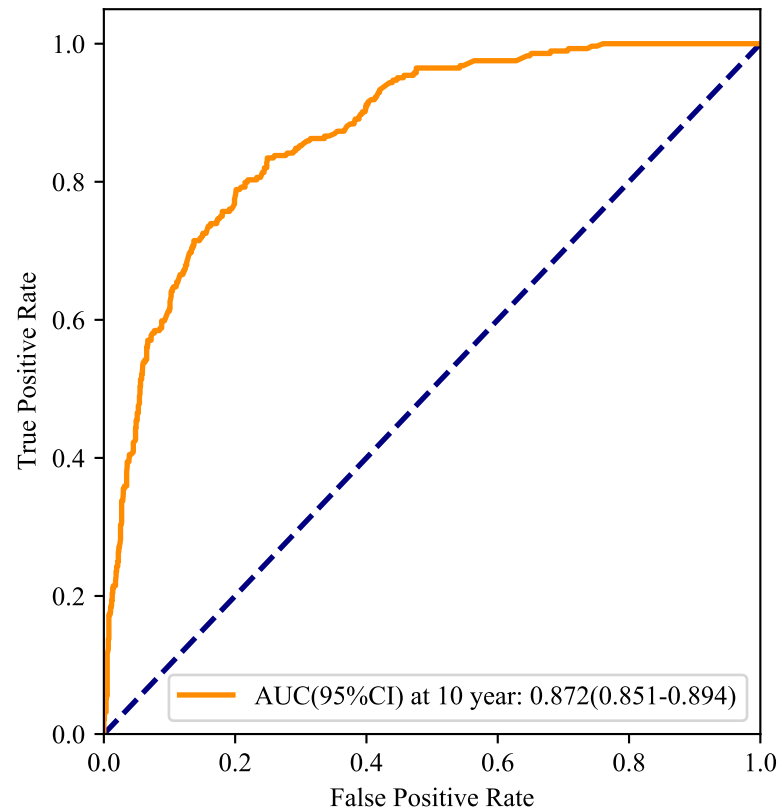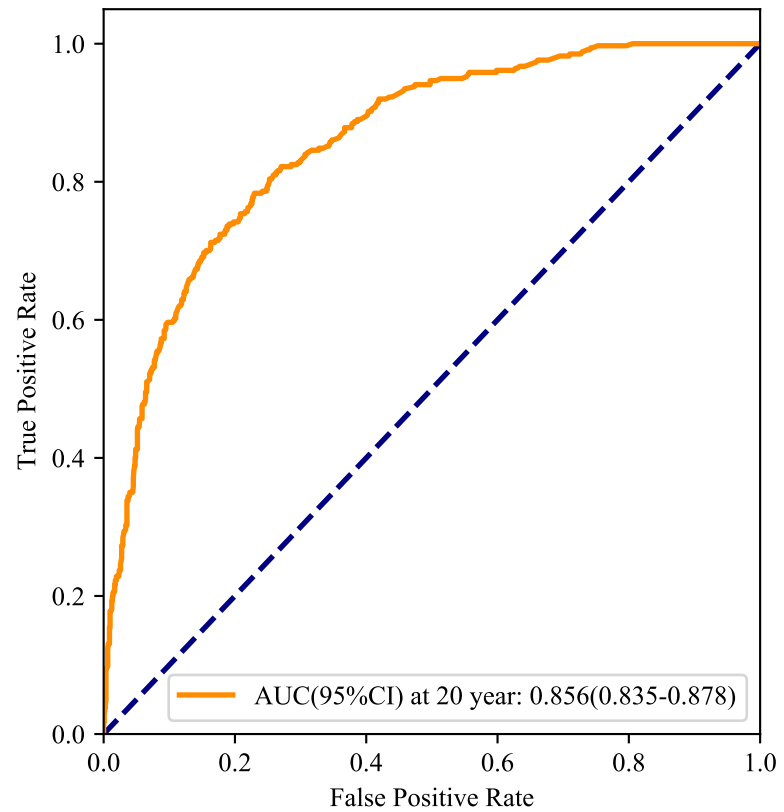

Supplement: Supplementary file 3 [file Datasheet3.pdf]

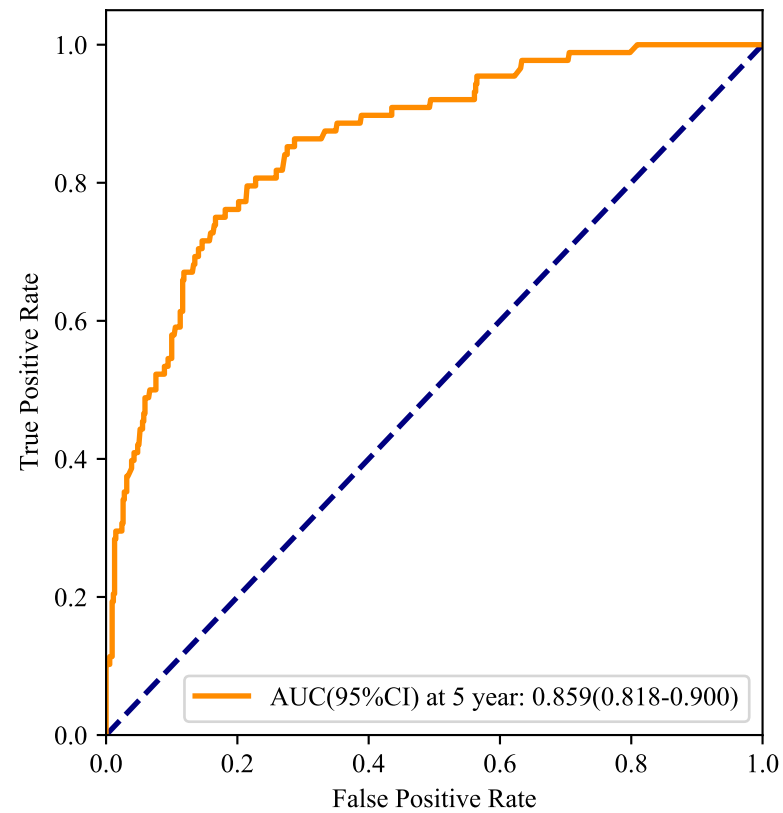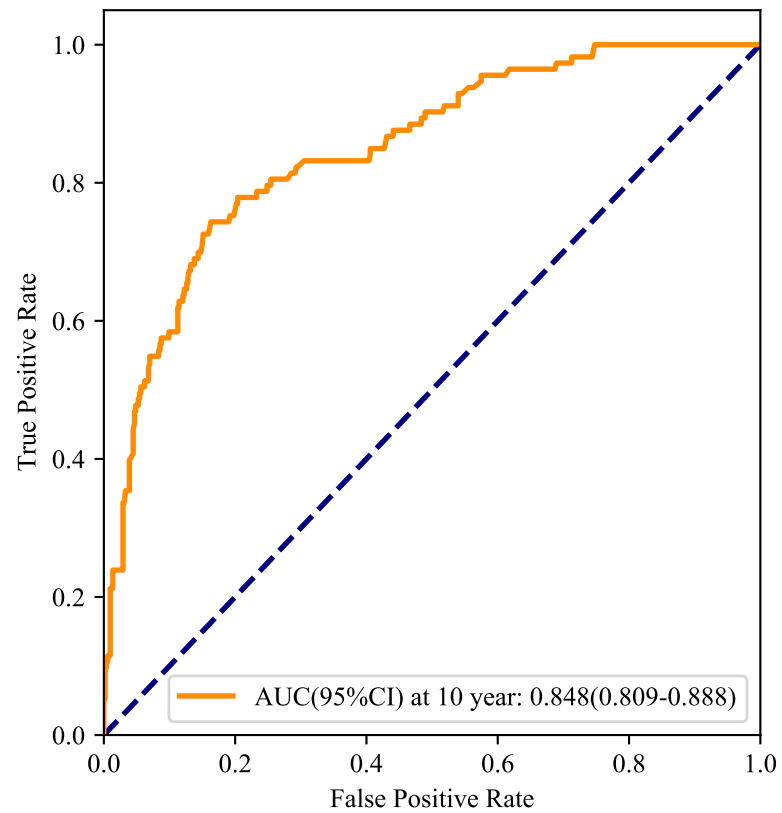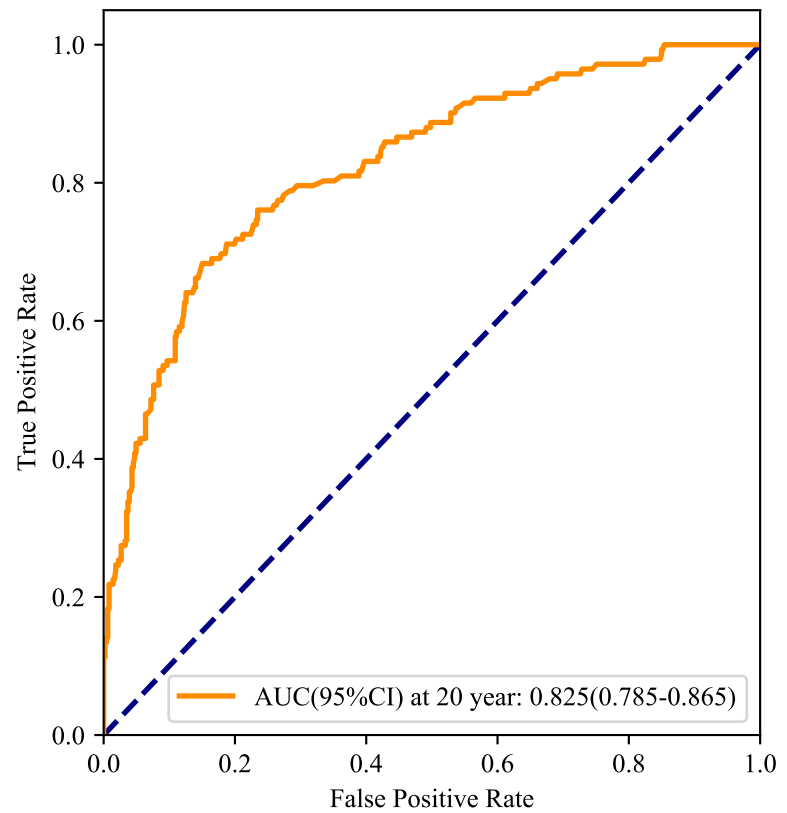

Supplement: Supplementary file 4 [file Datasheet4.pdf]

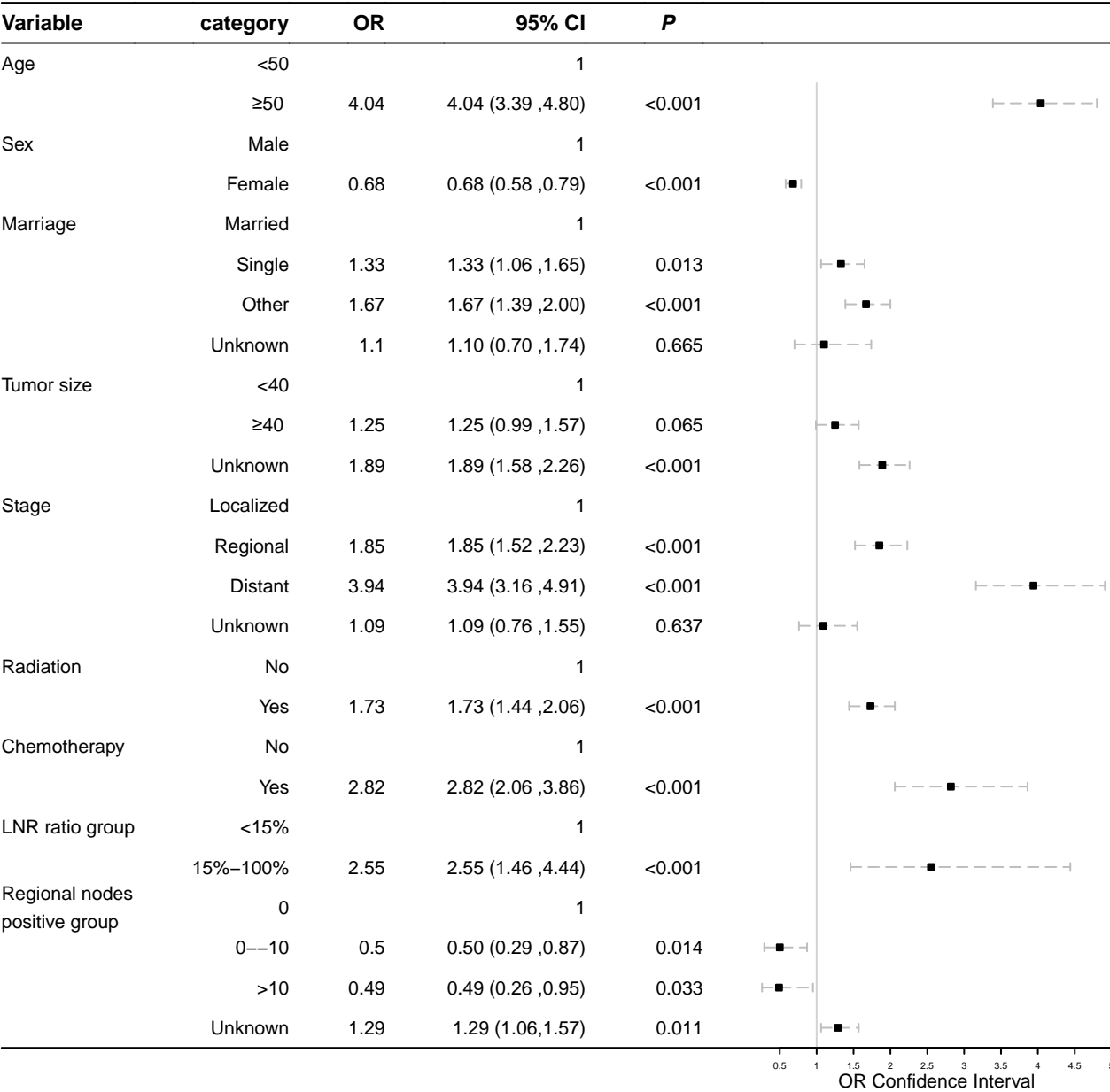

Supplement: Supplementary file 5 [file Datasheet5.pdf]

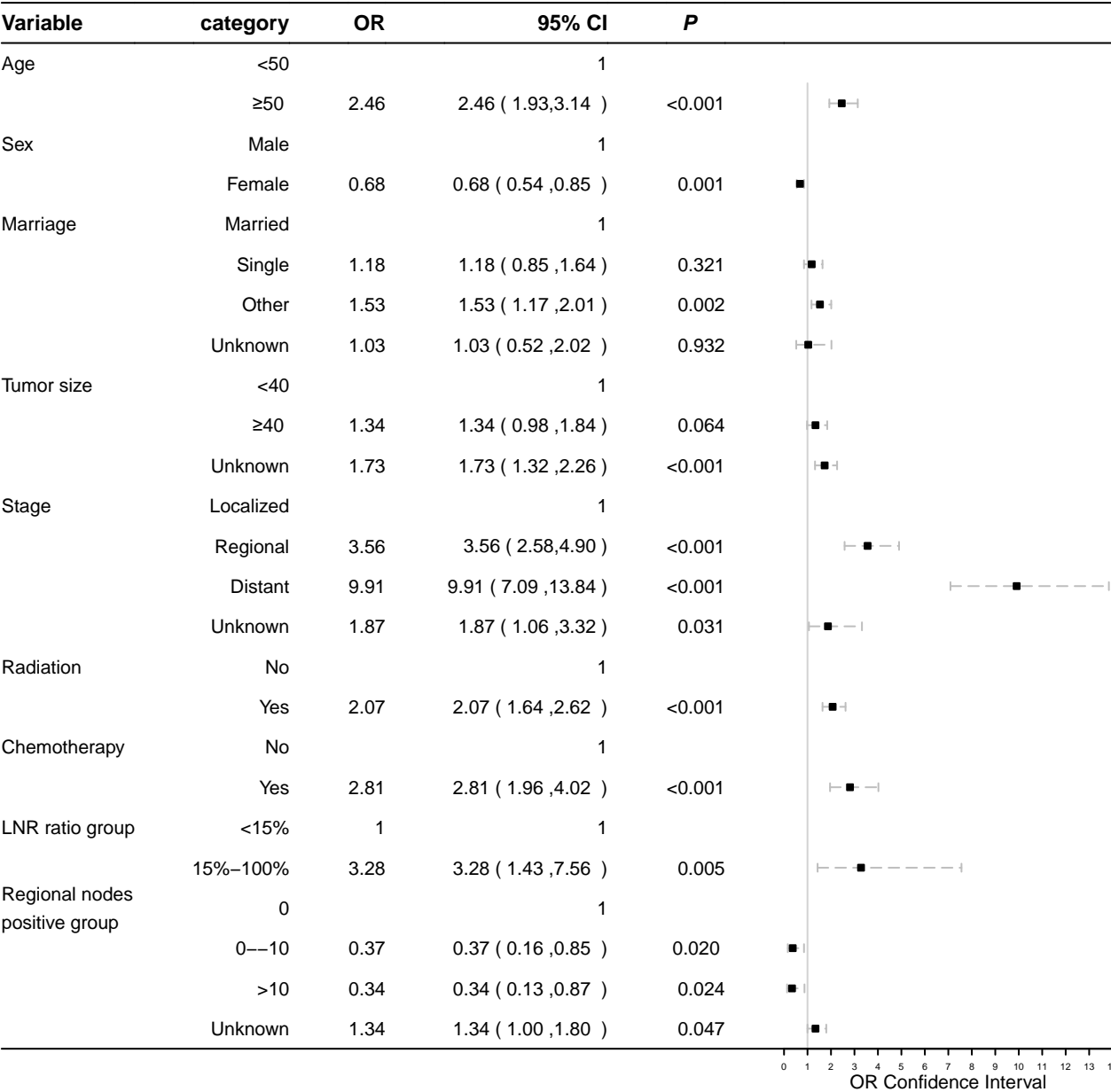

Supplement: Supplementary file 6 [file Datasheet6.pdf]
